# Ginger supplementation for the treatment of non-alcoholic fatty liver disease: a meta-analysis of randomized controlled trials

**DOI:** 10.4314/ahs.v23i1.65

**Published:** 2023-03

**Authors:** Qun Zhou, Ying Peng, Fangyuan Chen, Jianbo Dai

**Affiliations:** 1 Health management center, Chongqing General Hospital, China; 2 Internal medicine, Chongqing Nan'an District People's Hospital; 3 Department of general surgery, Chongqing Nan'an District People's Hospital

**Keywords:** ginger, non-alcoholic fatty liver disease, randomized controlled trials, meta-analysis

## Abstract

**Introduction:**

The efficacy of ginger supplementation remains controversial for non-alcoholic fatty liver disease. We conduct this meta-analysis to explore the influence of ginger supplementation versus placebo on the treatment of non-alcoholic fatty liver disease.

**Methods:**

We have searched PubMed, EMbase, Web of science, EBSCO, and Cochrane library databases through November 2021 and included randomized controlled trials (RCTs) assessing the efficacy of ginger supplementation versus placebo for non-alcoholic fatty liver disease. This meta-analysis was performed using the random-effect model.

**Results:**

Four RCTs involving 177 patients were included in the meta-analysis. Overall, compared with non-alcoholic fatty liver disease, ginger supplementation was associated with significantly reduced alanine aminotransferase (ALT, standard mean difference (SMD)=-0.43; 95% confidence interval [CI]=-0.85 to -0.02; P=0.04), homeostatic Model Assessment of Insulin Resistance (HOMA-IR, SMD=-1.14; 95% CI=-2.05 to -0.22; P=0.02), but revealed no obvious impact on aspartate-aminotransferase (AST, SMD=-0.66; 95% CI=-0.81 to 2.12; P=0.38), total cholesterol (SMD=-0.33; 95% CI=-0.67 to 0.02; P=0.06), low density lipoprotein (LDL, SMD=-0.30; 95% CI=-0.64 to 0.04; P=0.08) or body mass index (BMI, SMD=0; 95% CI=-0.41 to 0.40; P=0.99).

**Conclusions:**

Ginger supplementation benefits to treat non-alcoholic fatty liver disease.

## Introduction

Non-alcoholic fatty liver disease was identified by Ludwig in 1980 and accumulation of more than 5% triglyceride in hepatic parenchyma without notable alcohol consumption is defined as non-alcoholic fatty liver disease, which has become one global public health problem^1^–^4^. This disease is featured by a wide range of liver disorders from hepatocellular steatosis to more severe non-alcoholic steatohepatitis, which may progress to hepatic fibrosis and cirrhosis^5^–^7^.

There is still lack of effective treatment for non-alcoholic fatty liver disease, and new therapeutic approaches should be developed to improve the treatment efficacy^8^. Diets containing antioxidants and anti-inflammatory agents showed some benefits in the treating of non-alcoholic fatty liver disease^9^–^11^. Ginger was widely used worldwide as a spice, and demonstrated some efficacy to treat neurological diseases, diabetes, rheumatism, gingivitis, toothache, asthma, constipation, maldigestion, nausea and vomiting because of its roles in immune regulator, tumor inhibition, anti-inflammation, anti-apoptotic and antiemetic effects. There have been more than 40 antioxidant compounds that are identified in ginger^12^. Ginger family was reported to suppress nuclear factor-kappa B (NF-κB), which was the key protein complex for cytokine production and cellular responses to stimuli such as inflammatory cytokines and oxidative stress^13^–^15^.

In patients with non-alcoholic fatty liver disease, ginger supplement resulted in significant improvement in alanine aminotransferase (ALT), total cholesterol, low-density lipoprotein (LDL) and insulin resistance index (HOMA) compared to placebo 16. However, the benefit of ginger supplementation for non-alcoholic fatty liver disease has not been well established. Recently, several studies on the topic have been published, and the results were conflicting^16^–^18^. With accumulating evidence, we therefore searched for eligible randomized controlled trials (RCTs) assessing the efficacy of ginger supplementation versus placebo for non-alcoholic fatty liver disease and perform this meta-analysis using the random-effect model. Sensitivity analysis was also conducted. These aimed to explore the efficacy of ginger supplementation versus placebo for non-alcoholic fatty liver disease.

## Materials and methods

Ethical approval and patient consent were not required because this was a meta-analysis of previously published studies. This meta-analysis was conducted and reported in adherence to PRISMA (Preferred Reporting Items for Systematic Reviews and Meta-Analyses) 19, 20.

### Search strategy and study selection

Two investigators have independently searched the following databases (inception to November 2021): PubMed, EMbase, Web of science, EBSCO and Cochrane library databases. The electronic search strategy was conducted using the following keywords: “non-alcoholic fatty liver disease” OR “steatohepatitis” AND “ginger”. The inclusive selection criteria were as follows: (i) patients were diagnosed with non-alcoholic fatty liver disease; (ii) intervention treatments were ginger supplementation versus placebo; (iii) study design was RCT.

### Data extraction and outcome measures

We extracted the following information: author, number of patients, age, female, weight, body mass index and detail methods in each group etc. Data were extracted independently by two investigators, and discrepancies were resolved by consensus. The primary outcomes were alanine aminotransferase (ALT) and aspartate-aminotransferase (AST). Secondary outcomes included homeostatic Model Assessment of Insulin Resistance (HOMA-IR), total cholesterol, low density lipoprotein (LDL) and body mass index (BMI).

### Quality assessment in individual studies

Methodological quality of the included studies was independently evaluated using the modified Jadad scale 20,21. There were three items for Jadad scale: randomization (0-2 points), blinding (0-2 points), dropouts and withdrawals (0-1 points). The score of Jadad Scale varied from 0 to 5 points. An article with Jadad score≤2 had low quality. If the Jadad score≥3, the study was thought to have high quality 22, 23.

### Statistical analysis

We estimated the standard mean difference (SMD) with 95% confidence interval (CI) for continuous outcomes. The random-effects model was used regardless of heterogeneity. Heterogeneity was reported using the I2 statistic, and I2 > 50% indicated significant heterogeneity^24^. Whenever significant heterogeneity was present, we searched for potential sources of heterogeneity via omitting one study in turn for the meta-analysis or performing subgroup analysis. All statistical analyses were performed using Review Manager Version 5.3 (The Cochrane Collaboration, Software Update, Oxford, UK).

## Results

### Literature search, study characteristics and quality assessment

**Figure 1:**
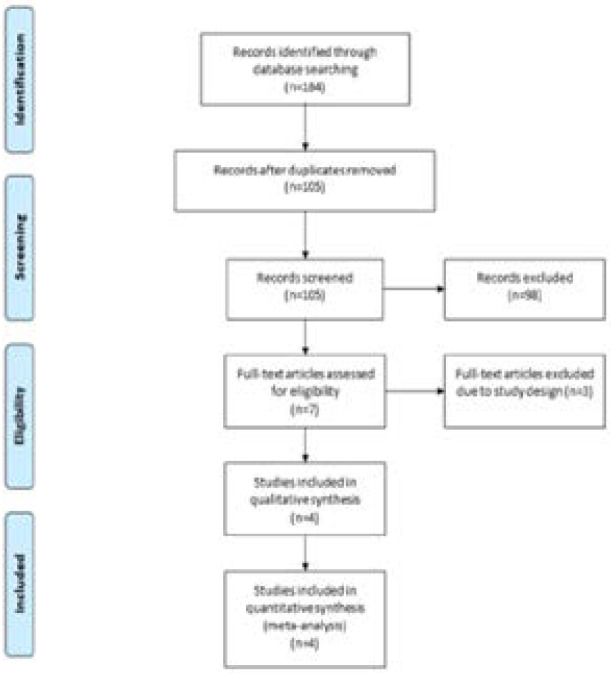
Flow diagram of study searching and selection process.

A detailed flowchart of the search and selection results was presented in Figure 1. 184 potentially relevant articles were identified initially and four RCTs were finally included in the meta-analysis 16–18, 25. The baseline characteristics of four eligible RCTs were summarized in Table 1. The four studies were published between 2016 and 2020, and total sample size was 177. The doses of ginger supplementation ranged from 1000 mg daily to 1500 mg daily. Two studies reported the same patient sample with different outcomes. Among the four studies included here, three studies reported ALT and AST 16, 18, 25, three studies reported HOMA-IR 16, 17, 25, two studies reported total cholesterol and LDL 16, 17 and two studies reported BMI^16^,^18^. Jadad scores of the four included studies varied from 3 to 4, and all four studies had high quality.

**Table 1:** Characteristics of included studies

NO.	Author, year	Ginger group						Control group						Jada scores
		Number	Age (years)	Female (n)	Weight (kg)	Body mass index (kg/m2)	Methods	Number	Age (years)	Female (n)	Weight (kg)	Body mass index (kg/m2)	Methods	
1	Rafie 2020	23	50.04 ± 10.26	13	-	31.70±3.75	1500 mg ginger powder daily for 12 weeks	23	47.95 ± 9.24	13	-	30.94±1.98	placebo	4
2	Daneshi-Maskooni 2019	43	45.5±8.9	16	85.2±11.3	30.5±2.4	two 500 mg capsules (green cardamom from ginger family) three times per day for 3 months	44	45.0±7.7	17	88.6±13.2	30.7±3.2	placebo	4
3	Daneshi-Maskooni 2018	43	45.5±8.9	16	85.2±11.3	30.5±2.4	two 500 mg capsules (green cardamom from ginger family) three times per day for 3 months	44	45.0±7.7	17	88.6±13.2	30.7±3.2	placebo	4
4	Rahimlou 2016	23	45.45±2.31	11	86.47±3.59	30.55±0.95	500 mg ginger capsules twice daily for 12 weeks	21	45±2.14	13	81.38±2.96	31.53±0.47	placebo	3

### Primary outcomes: ALT and AST

**Figure 2:**
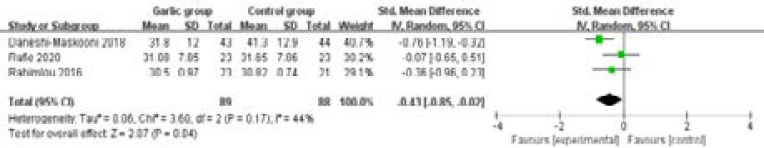
Forest plot for the meta-analysis of ALT.

**Figure 3:**
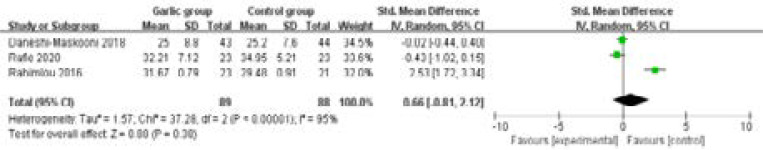
Forest plot for the meta-analysis of AST.

These outcome data were analysed with the random-effects model, and compared to control group for non-alcoholic fatty liver disease, ginger supplementation was associated with significantly reduced ALT (SMD=-0.43; 95% CI=-0.85 to -0.02; P=0.04) with low heterogeneity among the studies (I2=44%, heterogeneity P=0.17, Figure 2), but showed no obvious influence on AST (SMD=-0.66; 95% CI=-0.81 to 2.12; P=0.38) with significant heterogeneity among the studies (I2=95%, heterogeneity P=0.38, Figure 3).

### Sensitivity analysis

Significant heterogeneity was observed among the included studies for the AST. As shown in Figure 3, the study conducted by Rahimlou showed the results that were almost out of range of the others and probably contributed to the heterogeneity 25. After excluding this study, the results suggested that ginger supplementation also demonstrated no significant impact on AST (SMD=-0.17; 95% CI=-0.56 to 0.21; P=0.37), and low heterogeneity remained (I2=19%, P=0.27).

### Secondary outcomes

**Figure 4:**
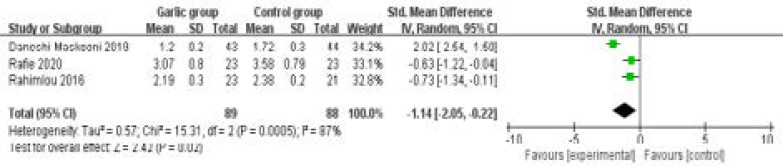
Forest plot for the meta-analysis of HOMA-IR

**Figure 5:**
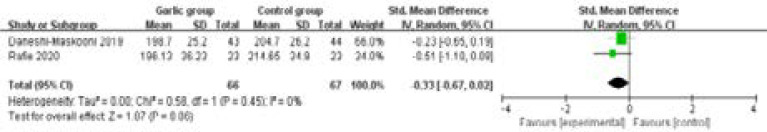
Forest plot for the meta-analysis of total cholesterol.

**Figure 6:**
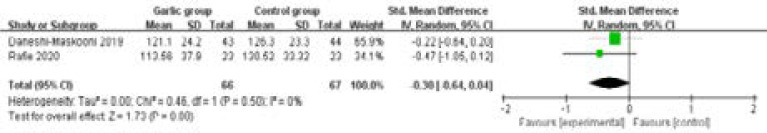
Forest plot for the meta-analysis of LDL.

**Figure 7:**
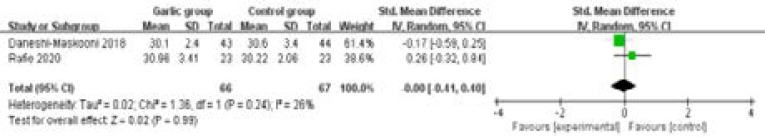
Forest plot for the meta-analysis of BMI.

In comparison with control group for control group for non-alcoholic fatty liver disease, ginger supplementation substantially reduced HOMA-IR (SMD=-1.14; 95% CI=-2.05 to -0.22; P=0.02; Figure 4), but unravelled no obvious influence on total cholesterol (SMD=-0.33; 95% CI=-0.67 to 0.02; P=0.06; Figure 5), LDL (SMD=-0.30; 95% CI=-0.64 to 0.04; P=0.08; Figure 6) or BMI (SMD=0; 95% CI=-0.41 to 0.40; P=0.99; Figure 7).

## Discussion

Inhibition of inflammatory and oxidative stress factors in patients with non-alcoholic fatty liver disease plays an important role in slowing the rate of progression and reducing the risk of cardiovascular disorders 26–28. Ginger has potential antioxidant activities due to its polyphenolic compounds as gingerol and curcumin, and thus inhibits lipid peroxidation 29. Our meta-analysis included four eligible RCTs and 177 patients, and the results confirmed that ginger supplementation exerted important beneficial effect on hepatic function and insulin resistance for non-alcoholic fatty liver disease, as evidenced by the significantly reduced ALT and HOMA-IR.

Two-hit hypothesis are widely accepted to cause non-alcoholic fatty liver disease. First hit is defined as insulin resistance that stimulates the synthesis of liver fatty acids and accumulation of fat in the liver and steatosis, and the liver is more susceptible to the second hit (i.e. oxidative stress from different sources) 16. Insulin resistance was substantially improved by ginger supplementation in our study. Insulin resistance, severe disorders of lipid metabolism, oxidative stress and inflammation play important roles in the pathogenesis of non-alcoholic fatty liver disease, and thus potential therapeutic agents are designed to target one or some of these pathological events. The fundamental role of insulin resistance is found in fat accumulation of liver, and the increase in insulin sensitivity is very promising against non-alcoholic fatty liver disease^16^, ^30^–^32^.

Several reasons may account for the beneficial effect of ginger supplementation on non-alcoholic fatty liver disease. Firstly, ginger has substantial ability to improve insulin sensitivity to adipocytes 33. Secondly, biosynthesis of cholesterol can be inhibited and more the transformation of cholesterol to bile acids is promoted by ginger treatment 34. Thirdly, ginger can inhibit arachidonic acid metabolism with the suppression of cyclooxygenase and lipoxygenase enzymes, thereby acting as an anti-inflammatory agent 16.

Regarding the sensitivity analysis, significant heterogeneity remained for AST. After excluding the study conducted by Rahimlou et al. 25, only low heterogeneity was found (I2=19%, P=0.27), and this heterogeneity was caused by the substantial difference in baseline AST 25. Our meta-analysis also has some importantt limitations. Firstly, our analysis is based on four RCTs, and all of them have a relatively small sample size (n<100). Overestimation of the treatment effect is more likely in smaller trials compared with larger samples. Secondly, although there is significant heterogeneity, different doses and forms of ginger supplementation may produce some bias. Thirdly, different etiologies and severity levels of non-alcoholic fatty liver disease may affect the efficacy assessment of ginger supplementation.

## Conclusions

Ginger supplementation provides additional benefits to treat non-alcoholic fatty liver disease.

## References

[1] Younossi ZM (2019). Non-alcoholic fatty liver disease - A global public health perspective. Journal of hepatology.

[2] Vuppalanchi R, Chalasani N (2009). Nonalcoholic fatty liver disease and nonalcoholic steatohepatitis: Selected practical issues in their evaluation and management. Hepatology (Baltimore, Md.).

[3] Neuschwander-Tetri BA (2017). Non-alcoholic fatty liver disease. BMC medicine.

[4] Katsiki N, Mikhailidis DP, Mantzoros CS (2016). Non-alcoholic fatty liver disease and dyslipidemia: An update, Metabolism. clinical and experimental.

[5] Marchesini G, Bugianesi E, Forlani G, Cerrelli F, Lenzi M, Manini R, Natale S, Vanni E, Villanova N, Melchionda N, Rizzetto M (2003). Nonalcoholic fatty liver, steatohepatitis, and the metabolic syndrome. Hepatology (Baltimore, Md.).

[6] Bedossa P (2017). Pathology of non-alcoholic fatty liver disease. Liver international :official journal of the International Association for the Study of the Liver.

[7] Vilar-Gomez E, Chalasani N (2018). Non-invasive assessment of non-alcoholic fatty liver disease: Clinical prediction rules and blood-based biomarkers. Journal of hepatology.

[8] Singh S, Osna NA, Kharbanda KK (2017). Treatment options for alcoholic and non-alcoholic fatty liver disease: A review. World journal of gastroenterology.

[9] Chen H, Huang W, Huang X, Liang S, Gecceh E, H OS, Khani V, Jiang X (2020). Effects of green coffee bean extract on C-reactive protein levels: A systematic review and meta-analysis of randomized controlled trials. Complementary therapies in medicine.

[10] Abbasnezhad A, Choghakhori R, Kashkooli S, Alipour M, Asbaghi O, Mohammadi R (2019). Effect of L-carnitine on liver enzymes and biochemical factors in hepatic encephalopathy: A systematic review and meta-analysis. Journal of gastroenterology and hepatology.

[11] Mahdavinia M, Alizadeh S, Raesi A, Vanani, Dehghani MA, Shirani M, Alipour M, Shahmohammadi HA, Rafiei Asl S (2019). Effects of quercetin on bisphenol A-induced mitochondrial toxicity in rat liver. Iranian journal of basic medical sciences.

[12] Ali BH, Blunden G, Tanira MO, Nemmar A (2008). Some phytochemical, pharmacological and toxicological properties of ginger (Zingiber officinale Roscoe): a review of recent research. Food and chemical toxicology: an international journal published for the British Industrial Biological Research Association.

[13] Gilmore TD (2006). Introduction to NF-kappaB: players, pathways, perspectives. Oncogene.

[14] Brglez Mojzer E, Knez Hrnčič M, Škerget M, Knez Ž, Bren U (2016). Polyphenols: Extraction Methods, Antioxidative Action, Bioavailability and Anticarcinogenic Effects, Molecules (Basel, Switzerland).

[15] Hämäläinen M, Nieminen R, Vuorela P, Heinonen M, Moilanen E (2007). Anti-inflammatory effects of flavonoids: genistein, kaempferol, quercetin, and daidzein inhibit STAT-1 and NF-kappaB activations, whereas flavone, isorhamnetin, naringenin, and pelargonidin inhibit only NF-kappaB activation along with their inhibitory effect on iNOS expression and NO production in activated macrophages. Mediators of inflammation.

[16] Rafie R, Hosseini SA, Hajiani E, Saki Malehi A, Mard SA (2020). Effect of Ginger Powder Supplementation in Patients with Non-Alcoholic. Fatty Liver Disease: A Randomized Clinical Trial, Clinical and experimental gastroenterology.

[17] Daneshi-Maskooni M, Keshavarz SA, Qorbani M, Mansouri S, Alavian SM, Badri-Fariman M, Jazayeri-Tehrani SA, Sotoudeh G (2019). Green cardamom supplementation improves serum irisin, glucose indices, and lipid profiles in overweight or obese non-alcoholic fatty liver disease patients: a double-blind randomized placebo-controlled clinical trial. BMC complementary and alternative medicine.

[18] Daneshi-Maskooni M, Keshavarz SA, Qorbani M, Mansouri S, Alavian SM, Badri-Fariman M, Jazayeri-Tehrani SA, Sotoudeh G (2018). Green cardamom increases Sirtuin-1 and reduces inflammation in overweight or obese patients with non-alcoholic fatty liver disease: a double-blind randomized placebo-controlled clinical trial. Nutrition & metabolism.

[19] Moher D, Liberati A, Tetzlaff J, Altman DG, P. Group (2009). Preferred reporting items for systematic reviews and meta-analyses: the PRISMA statement. Journal of clinical epidemiology.

[20] He B, Zhao J-Q, Zhang M-Z, Quan Z-X (2021). Zoledronic acid and fracture risk: a meta-analysis of 12 randomized controlled trials. Eur Rev Med Pharmacol Sci.

[21] Jadad AR, Moore RA, Carroll D, Jenkinson C, Reynolds DJM, Gavaghan DJ, McQuay HJ (1996). Assessing the quality of reports of randomized clinical trials: Is blinding necessary?. Controlled Clinical Trials.

[22] Kjaergard LL, Villumsen J, Gluud C (2001). Reported Methodologic Quality and Discrepancies between Large and Small Randomized Trials in Meta-Analyses. Annals of Internal Medicine.

[23] Zhao J, Huang W, Zhang S, Xu J, Xue W, He B, Zhang Y (2020). Efficacy of Glutathione for Patients with Cystic Fibrosis: A Meta-analysis of Randomized-Controlled Studies. Am J Rhinol Allergy.

[24] Higgins JP, Thompson SG (2002). Quantifying heterogeneity in a meta-analysis. Statistics in medicine.

[25] Rahimlou M, Yari Z, Hekmatdoost A, Alavian SM, Keshavarz SA (2016). Ginger Supplementation in Nonalcoholic Fatty Liver Disease: A Randomized, Double-Blind, Placebo-Controlled Pilot Study. Hepatitis monthly.

[26] Mukhopadhyay P, Horváth B, Rajesh M, Varga ZV, Gariani K, Ryu D, Cao Z, Holovac E, Park O, Zhou Z, Xu MJ, Wang W, Godlewski G, Paloczi J, Nemeth BT, Persidsky Y, Liaudet L, Haskó G, Bai P, Boulares AH, Auwerx J, Gao B, Pacher P (2017). PARP inhibition protects against alcoholic and non-alcoholic steatohepatitis. Journal of hepatology.

[27] Hwangbo H, Kim MY, Ji SY, Kim SY, Lee H, Kim GY, Park C, Keum YS, Hong SH, Cheong J, Choi YH (2020). Auranofin Attenuates Non-Alcoholic Fatty Liver Disease by Suppressing Lipid Accumulation and NLRP3 Inflammasome-Mediated Hepatic Inflammation In Vivo and In Vitro. Antioxidants (Basel, Switzerland).

[28] Shen B, Zhao C, Wang Y, Peng Y, Cheng J, Li Z, Wu L, Jin M, Feng H (2019). Aucubin inhibited lipid accumulation and oxidative stress via Nrf2/HO-1 and AMPK signalling pathways. Journal of cellular and molecular medicine.

[29] Stoilova I, Krastanov A, Stoyanova A, Denev P, Gargova S (2007). Antioxidant activity of a ginger extract (Zingiber officinale). Food Chemistry.

[30] Spahis S, Alvarez F, Ahmed N, Dubois J, Jalbout R, Paganelli M, Grzywacz K, Delvin E, Peretti N, Levy E (2018). Non-alcoholic fatty liver disease severity and metabolic complications in obese children: impact of omega-3 fatty acids. The Journal of nutritional biochemistry.

[31] Arroyave-Ospina JC, Wu Z, Geng Y, Moshage H (2021). Role of Oxidative Stress in the Pathogenesis of Non-Alcoholic Fatty Liver Disease: Implications for Prevention and Therapy. Antioxidants (Basel, Switzerland).

[32] Farzanegi P, Dana A, Ebrahimpoor Z, Asadi M, Azarbayjani MA (2019). Mechanisms of beneficial effects of exercise training on non-alcoholic fatty liver disease (NAFLD): Roles of oxidative stress and inflammation. European journal of sport science.

[33] Sahebkar A (2011). Potential efficacy of ginger as a natural supplement for nonalcoholic fatty liver disease. World journal of gastroenterology.

[34] Verma SK, Singh M, Jain P, Bordia A (2004). Protective effect of ginger, Zingiber officinale Rosc on experimental atherosclerosis in rabbits. Indian journal of experimental biology.

